# Investigations on the Particle Fouling and Backwash Efficiency During Microplastic Microfiltration–Particle Size Aspects

**DOI:** 10.3390/membranes15090272

**Published:** 2025-09-09

**Authors:** Saeedeh Saremi, Leonie Marie Scheer, Gerhard Braun, Marcus Koch, Markus Gallei, Matthias Faust

**Affiliations:** 1 Institute for Physical Process Technology, Saarland University of Applied Sciences, 66117 Saarbrücken, Germany; saeedeh.saremi@htwsaar.de (S.S.); gerhard.braun@htwsaar.de (G.B.); marcus.koch@htwsaar.de (M.K.); 2 Polymer Chemistry, Saarland University, 66123 Saarbrücken, Germany; markus.gallei@uni-saarland.de; 3 Saarene, Saarland Center for Energy Materials and Sustainability, Saarland University, 66123 Saarbrücken, Germany

**Keywords:** microplastic separation, microfiltration, particle-fouling analysis, water treatment process

## Abstract

The characteristics of polystyrene (PS) microplastic (MP) microfiltration by a cellulose acetate (CA) membrane were investigated within this study. Particle sizes and pore sizes were selected in a comparable range in order to challenge the dead-end microfiltration. Backwashing experiments round up the investigations. Microfiltration characteristics and particle size measurements, as well as a particle fouling analysis by different methods, were applied in the study in order to provide a comprehensive picture of particle deposition and particle fouling structuring. The particle removal efficiency was particle-size-dependent, and especially small particles were further reduced during the proceeding filtration, while the larger particles were already removed within the first minutes of filtration. This observation was attributed to the pore blocking (internal and/or complete) and build-up of the filter cake. The difference in the particle-fouling structure at low and elevated filtration pressure significantly influences the backwashing efficiency. The particle fouling resulting from low-pressure filtration was completely removed due to the backwashing procedure applied, while an increased filtration pressure resulted in a different particle-fouling structure, which negatively influenced the backwashing efficiency. This knowledge of the formation and structure of the MP particle fouling and its removal by backwashing is a prerequisite for further process development.

## 1. Introduction

The increasing pollution of the environment by microplastics (MPS) is a global challenge that endangers aquatic ecosystems and potentially compromises the safety of drinking-water resources. The tiny plastic particles enter the environment from a wide variety of sources and via multiple pathways and are now ubiquitously detectable in water bodies and other ecosystems [1,2].

Numerous articles regarding the origin and measurement of microplastics (1 µm–5 mm) in water, as well as possible removal strategies for microplastics from water, were published within the last decade. Most of the papers focus on the hazardous properties of microplastics and/or nanoplastics and on engineering processes and cleaning strategies, e.g., sedimentation, coagulation/flocculation, degradation, or filtration [2,3,4,5,6,7,8]. Filtration, and especially membrane filtration, seems to be a promising separation technology for the removal of microplastics from drinking water, pre-treated water from industry processes, or pre-cleaned water from sewage plants [2,3,4,5,6,7,9,10,11,12,13,14,15,16]. Nearly all studies in the literature focus on the environmental technology aspects, such as material, average size, associated biofouling, or efficiency of removal technologies. However, there are only a few studies that analyze filtration and particle-fouling characteristics experimentally from a particle-engineering perspective.

For example, Enfrin et al. investigated the usage of ultrafiltration membranes for the removal of micro/nanoplastics (NP) in detail. They found that hydrophilic membranes have a lower adsorption rate of NP/MP compared to hydrophobic membranes [17,18]. Investigations on the usage and efficiency of gas scouring for the reduction of NP/MP fouling were also performed by Enfrin et al. [18]. Experimental investigations regarding the usage of microfiltration membranes for the separation of microplastics were performed by Pizzichetti et al. [19,20]. The results showed that the microfiltration membranes are, in principle, suitable for the controlled removal of microplastics from water. Pizzichetti et al. used plastic particles in a size range between 20 and 300 µm. Microfiltration membranes with a nominal pore size of 5 µm were used during these experiments and received nearly complete removal of the particles [19,20]. The results confirmed that microfiltration could be a suitable tool for the removal of microplastics in many applications. Pizzichetti et al. also studied the PS and PA (polyamide) particle fouling on a CA membrane. Due to the use of relatively large particles compared to the small CA membrane pores, a complete pore blockage followed by a build-up of a filter cake was observed and evaluated in detail during their investigations [20].

Cellulose acetate (CA) is a sustainable polymer that is used in a number of applications, e.g., separation membranes, packaging films, medical devices, or composite materials. Besides its low costs, one of the main advantages of CA is its relatively easy recycling in comparison to most other polymers [21]. CA membranes are produced for nearly all membrane operations, e.g., reverse osmosis, nanofiltration, ultrafiltration, and microfiltration [22].

The selection and the efficiency of a filtration system usually depend on the feed stream composition and on the required filtrate characteristics.

Due to sustainability reasons, the membrane material, area, pore size, and thickness are usually selected by the operator depending on, e.g., the required flux, the operation time, the fouling mechanism, and possible membrane-cleaning opportunities [23].

In large-scale microfiltration operations, the pore size is usually selected to be smaller than the particle size. However, especially in applications in environmental technology, the particle size can vary. In order to choose an optimized set-up for our investigations, the membrane pores are in the same size range as the filtered particles, which challenges the membrane. This approach could be a powerful tool to gain a better understanding of microplastics deposition and particle fouling during filtration and gives important hints on the filtration mechanism, which is a prerequisite for possible anti-fouling membrane optimizations [24]. Backwashing is a common method in order to remove deposits on and in the membrane. Apart from flushing, backwashing is a physical treatment that requires only elevated process conditions and suitable process design. No additional hazardous chemicals are necessary for this method. Therefore, backwashing is a sustainable way to adjust and increase the microfiltration membranes’ operation time. It is a prerequisite for the scale-up of many membrane applications. Backwashing-efficiency tests round off the investigations because the backwash possibility is often a prerequisite for the usage of specific membranes in the field.

## 2. Materials and Methods

Polystyrene particles were provided by Coating Products OHZ (Osterholz-Scharmbeck, Germany). Very narrowly distributed polystyrene (PS) MS-5 FHC microparticles (1–20 µm) with a median diameter of d_50_._3_ 4–6 µm were selected for our investigations in order to challenge the CA microfiltration membrane (Sartorius Stadim Biotech GmbH, Goettingen, Germany), which is characterized by a pore size of 5 µm given by the producer. For validation, the median pore size of the surface pores was calculated to be d_50_._0_ 4.0 µm (scanning electron microscopy, see Supplementary Information). This particle–membrane combination enables the study of the pore-blocking mechanism as well as the structuring of the top layer (filter cake) during filtration, which is dependent on the trans-membrane pressure (TMP). The filtration efficiency was calculated via a comparison of the particle size distribution and the turbidity in the feed suspension and the filtrate (after defined time intervals) at different filtration pressures. In order to study the effect of the backwash, a second microfiltration test series was performed under equal conditions. Subsequent to the filtration experiment, the set-up was switched to backwash mode, and a 5 min backwash was carried out. The backwash efficiency was checked via the comparison of the initial pure water flux with the pure water flux after backwashing.

Besides the particle analysis and turbidity measurement in feed and filtrate, the fresh membranes, as well as the used membranes and the membranes after backwashing, were holistically investigated via light microscopy, SEM (scanning electron microscopy), contact angle measurement, and thermogravimetric analysis.

Thermogravimetric analysis (TGA) is a common method used to determine the thermal properties of materials (e.g., decomposition temperature). However, to our knowledge, there are only a few publications that focus on the usage of TGA for the investigation of membrane fouling [25,26]. Within these studies, a sample of the fouled membrane is dynamically heated in a N_2_ flow, while the mass of the sample is constantly recorded. Tay et al. [25] described the method for the investigation of membrane fouling in 2003. According to their observation, a difference in the decomposition temperatures of the membrane material (polymer) and the foulant (albumin in their experiments) is a prerequisite for the distinction between weight loss derived from the membrane and weight loss derived from the foulant during the TG analysis. Reactions between the foulant and the membrane must be excluded. Therefore, TGA measurements of the membrane material and of the foulant are required before the analysis of the fouled membrane. The publication by Kamarudin et al. [26] confirmed the suitability of TGA as a tool for the investigation of membrane fouling in 2022, presupposing that the decomposition temperatures of the materials deployed are different. Weight loss derived from the fouling and weight loss derived from the membrane were clearly observed during both studies. Due to the appearance of the thermogram, it was even possible to state which kind of fouling mechanism occurred during the prior filtration experiments. The two studies detected natural organic foulants, which implies the advantage that the fouling-decomposition temperature is very low in comparison to the decomposition temperature of the polymer membranes. During our investigations, we examined the potential usage of TGA for the analysis of microplastic fouling on membranes. We came to the conclusion that TGA could be a promising method to study fouling on inorganic membranes with high decomposition temperatures, which should be far above the decomposition temperature of the plastic particles. However, due to many advantages, it is more probable that polymeric membranes will be used for the separation of MPs in future applications. The elevated decomposition temperatures of the plastic particles, which are usually very close to the decomposition temperature of the polymeric membrane, make it very difficult to distinguish between a weight loss derived by the foulant or from the membrane. We investigated the decomposition characteristics of different MP materials and different membrane materials. In comparison to most membrane materials, CA is characterized by a relatively low decomposition temperature of less than 400 °C [27,28], while the foulant PS decomposes at around 400 °C or even higher temperatures [29,30,31]. Based on this research, we came to the conclusion that a detection of fouling by TGA could be feasible for the combination of the CA membrane and PS foulant.

### 2.1. Preparation of MP Particle–Water Suspensions

Previous to the filtration experiments, tap water was filtered through two microfiltration filter cartridges, followed by an ultrafiltration cartridge in order to provide comparable water conditions for the generation of the particle–water suspensions. The particle size distribution within the feed water was measured prior to each experiment. Homogeneous PS particle–feed water suspensions (5 mg PS/kg water) were generated by continuously stirring the PS water mixture for 30 min using a propeller agitator (Heidolph, Schwabach, Germany) at 500 RPM. The particle size distribution was measured by Klotz Syringe particle analysis system (Markus Klotz GmbH, Bad Liebenzell, Germany, LDS 30/30, equipped with a laser sensor, optical counting method, measurement range 0.9–139 µm, syringe size 10 mL) after different durations in order to ensure accurate and comparable feed suspensions for all filtration experiments. At least three measurements were applied for each sample. A magnetic stirrer integrated into the base of the particle measurement device ensures homogeneous particle distribution during the measurement. Data is recorded using the Protrend software (Version 3.3.1). SEM images of the PS particles were analyzed for verification of the particle measurement system results. For the turbidity measurement, samples were collected from the feed suspension and from the filtrates in three independent cuvettes at predefined time intervals (5 min, 30 min, and 60 min) and analyzed using the Hach 2100N device (Hach, Loveland, CO, USA). The measurement mode 0-1 NTU with a maximum resolution of 0.001 NTU was selected for the turbidity analysis.

### 2.2. Microfiltration Experiments and Backwash Procedure

**Figure 1:**
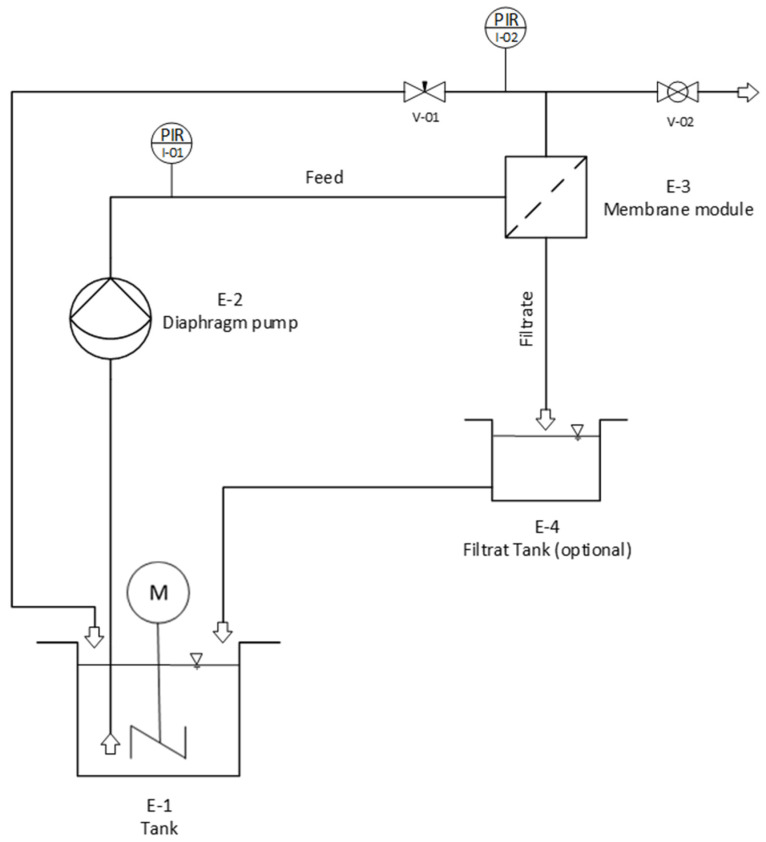
Flow sheet of the experimental filtration set-up.

A self-built experimental set-up was used for the dead-end microfiltration experiments (Figure 1). The flat sheet membranes were placed in a stainless steel filter holder (E-3, Sartorius Lab Instruments GmbH & Co. KG, Goettingen, Germany, model 16254, membrane diameter 47 mm, effective diameter 40 mm), which was equipped with an additional feed side needle valve (V-02) for the removal of air from the system (during the filling procedure). A volume of 2 L of the feed suspension (E-1) was continuously stirred by a magnetic stirrer in order to prevent any sedimentation. During dead-end operation, the needle valve (V-01) on the filtrate side remained closed. The feed stream was pumped through the membrane by a SHURflo diaphragm pump (E-2, Pentair Shurflo, Costa Mesa, USA, model 8015-114-111, which was controlled by a Conrad electronics laboratory power supply, 0–24 V, Conrad Electronics, Hirschau, Germany). Two pressure sensors (Hydac Electronic GmbH, Saarbruecken, Germany), in combination with a data-recording system (Hydac HMG 3000), ensure the online recording of the pressure, which was used to control the constant trans-membrane pressure during the experiment. After passage of the membrane, the filtrate was reintroduced into the feed suspension. The filtrate flux was measured after defined time intervals (5 min, 30 min, and 60 min) gravimetrically (E-4) and compared with the pure water flux of the respective membrane, which was measured prior to each experiment. At least three measurements were performed for each measurement point. Particle size distributions and turbidity in the filtrate were measured using a Klotz Syringe particle analysis system, LDS 30/30, and a Hach 2100N device. The mass removal efficiency (MRE%) as well as the particle removal efficiency (NpRE%) were calculated from the particle size distributions of the particles in the feed suspension and in the filtrate (mathematical equations are included in the Supplementary Information). The analysis of the particle size distributions and the calculation of the mass and particle number removal rates enable a valid evaluation of the membrane performance. The results are decisive for the optimization of filtration processes and offer starting points for further work.

The used membranes were carefully removed from the filter housing and dried an air atmosphere for one night before further investigations. The set-up was rinsed with clean water after each experiment, and all tubing was renewed before a new microfiltration experiment started.

For backwashing, it was necessary to reverse the water flow through the system at constant pumping conditions (24 V power supply) subsequent to the microfiltration. Therefore, the pump was installed on the filtrate side. The backwash was carried out for 5 min (0.44 bar), while the water was collected in an additional beaker. Subsequent to the completion of the backwash procedure, the configuration was transitioned back to the filtration mode, and the membrane’s pure water flow rate was evaluated gravimetrically. This flow was compared to the pure water flow prior to the filtration experiment in order to characterize the backwashing efficiency.

### 2.3. Characterization of the Particle Fouling

For visualization of the particles on top and in cross-sections of the membranes, light microscopy (LM) and scanning electron microscopy (SEM) were carried out. Additionally, water contact angle, Fourier Transform Infrared Spectroscopy (FTIR), and TGA measurements were performed in order to characterize the membrane particle fouling.

### 2.4. Microscopy

For light microscopy, the membranes were dried under ambient conditions and imaged in transmission mode using an Olympus CX 30 (Evident Corporation, Tokyo, Japan) equipped with a Jenoptik Progres C3 CCD camera (Jenoptik AG, Jena, Germany).

A JEOL JSM-6460LV scanning electron microscope (SEM) (JEOL Ltd., Tokyo, Japan) was used at 15 kV accelerating voltage in high vacuum. Secondary electron images of the top layer and cross-sections were obtained after coating the dried samples with gold by sputter deposition (Emitech K575X, Laughton, UK). Cross-sections of the samples were prepared by placing the membranes in a flat metallic cylinder filled with liquid ethanol, freezing the ethanol by dipping the metallic cylinder in a Dewar vessel filled with liquid nitrogen, and using a scalpel to cut the membranes embedded in frozen ethanol into two pieces. After warming up, the membrane pieces were dried on a tissue and placed on the side of a rectangular Al block using double-sided carbon tape to image the inner structure of the samples.

### 2.5. Water Contact Angle Measurement

To measure the water contact angle, a self-built contact angle goniometer was used, which consisted of an inclined Zeiss Standard 16 light microscope (Carl Zeiss AG, Oberkochen, Germany) with a horizontally movable sample stage and a Jenoptik Progres C3 CCD camera for lateral visualization of the water droplets. Therefore, a 5 µL water droplet was carefully placed on top of the membrane, the contact angle was measured after 10 s. ImageJ Ver. 1.54 was used to analyze the shape of the droplet using the plugin “drop_analysis”, and the water contact angle was determined.

### 2.6. Fourier Transform Infrared Spectroscopy (FTIR)

FTIR and micro-FTIR are common methods for the characterization of microplastics [32]. FTIR was performed with Bruker Alpha (Bruker Optics, Ettlingen, Germany) at wave numbers between 400 and 4000 cm^−1^ with a resolution better than 2 cm^−1^. A standard ATR (attenuated total reflection) method was used for the analysis.

### 2.7. Thermogravimetric Analysis (TGA)

The MP fouling was also determined via thermogravimetric analysis (TGA) using a Netzsch TG 209 (NETZSCH-Gerätebau GmbH, Selb, Germany). Accurate measurement conditions were achieved by an elaborate preparation before each analysis. The Al_2_O_3_ crucible was heated in air and subsequently in N_2_ up to 800 °C prior to each measurement. For calibration, the crucible was then heated in N_2_ atmosphere (100 mL/min) using a dynamic heating rate of 10 K/min from 20 to 800 °C, followed by the analysis of the sample under equal conditions. The weight loss was monitored, and DTG (differential thermogravimetric) curves were calculated. A detailed understanding of the thermal decomposition of the pure membrane and fouling materials is a prerequisite for the analysis of fouling by TGA. Especially for CA, structural changes can occur during treatment in an aqueous environment [33]. Therefore, TGA of CA membranes and PS particles, which were exposed to equal aqueous process conditions (without filtration), were also analyzed for comparison (Supplementary Information).

## 3. Results

### 3.1. Preparation of the Suspension

**Figure 2:**
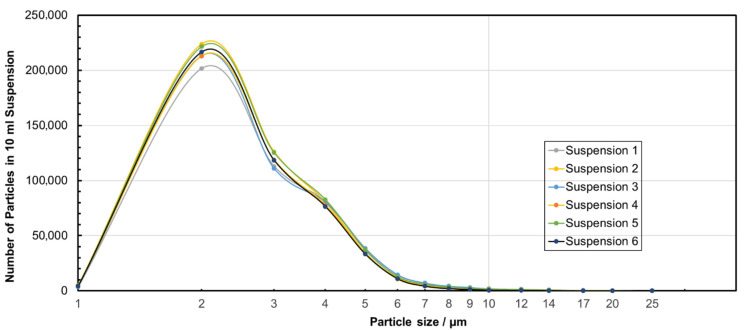
Particle size distributions of PS microplastic/water feed suspensions for the microfiltration experiments (lines are guides for the eyes).

Homogeneous microplastic/water feed suspensions are a prerequisite for filtration experiments at accurate test conditions. The number particle size distributions of six exemplary feed suspensions are given in Figure 2. Average particle sizes, standard deviation of the size distribution, and results of the turbidity measurements (Table 1) confirmed the generation of homogenous suspensions as well as the suitability of the preparation method. The median particle size d_50_._0_ was calculated to be 3.0 µm for the MP particles.

### 3.2. Microfiltration Experiments

For all filtration experiments, a significant decrease in the turbidity was measured after five minutes of filtration time. Taking into account the reference value and the standard deviations in the measurements, the turbidity values for all filtrates were 0.0 NTU. This result indicates that the particle concentration was already reduced to a very high extent after only five minutes of filtration time. As the turbidity measurement does not provide any detailed information about the remaining particle sizes in the filtrate, the filtration process is analyzed in more detail below using the particle size distributions.

**Figure 3:**
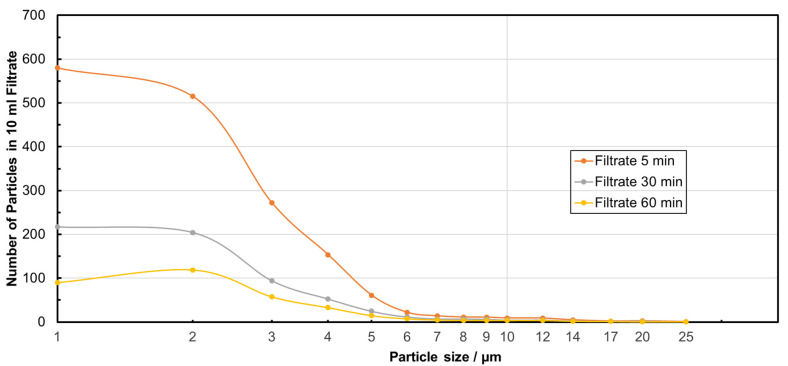
Particle size distributions of PS microplastic in filtrate after different filtration durations.

**Figure 4:**
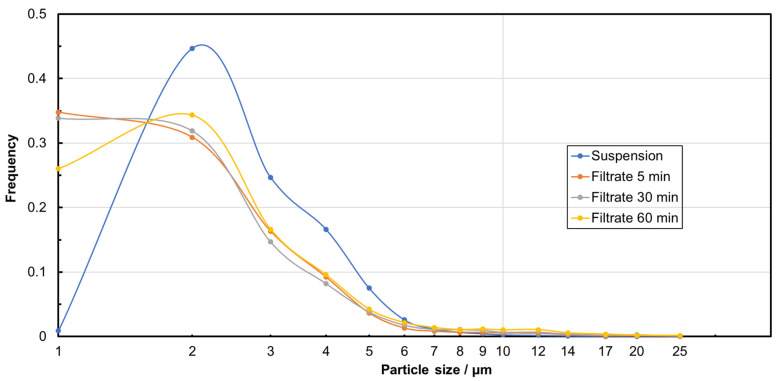
Comparison of the particle size distribution (frequency) of PS microplastic in the feed suspension and in the filtrate after different filtration durations.

As described in Section 2, both MRE% and NpRE% were calculated for each filtration experiment after 5, 30, and 60 min. Very high, filtration-time-dependent particle removal rates between 99 and 100% with very narrow standard deviations were achieved for all experiments, independent of the TMP applied (Table 2). The average values of the particle size classes in the filtrate for all tested TMP, but after different filtration times, are shown in Figure 3. In comparison to the feed suspension, a time-dependent decrease in the number of particles was detected. Particles smaller than the nominal pore size of the membrane were also removed in a remarkably high proportion, which indicates that a top layer (filter cake) formation and/or pore blockage significantly influenced the filtration mechanism. Taking into account the frequency of the particle sizes (Figure 4), it becomes obvious that the percentage of smaller particles (e.g., 1 µm) in the filtrate increased in comparison to the percentage in the feed suspension. The retention is therefore not initially the same for all particle sizes, so that smaller particles tend to remain in the filtrate, while larger particles are separated more quickly, which is characteristic of a filter cake. The calculation of the median particle sizes d_50_._0_ for the feed and for the filtrate after different filtration times confirms these observations (Table 2). The slight increase in the median particle size in the filtrate after 60 min could indicate that smaller particles are also retained more effectively in the further course of filtration due to the increasing filter cake. Overall, the two figures enable a comprehensive analysis of the filtration behavior by considering both absolute particle retention and changes in the size distribution.

**Figure 5:**
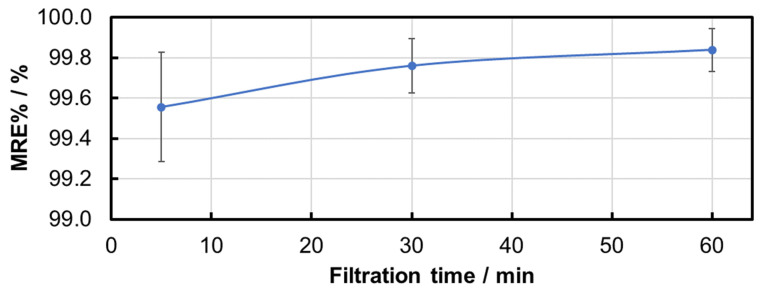
Time dependency of the average MRE% and standard deviation during all PS MP filtration experiments.

**Figure 6:**
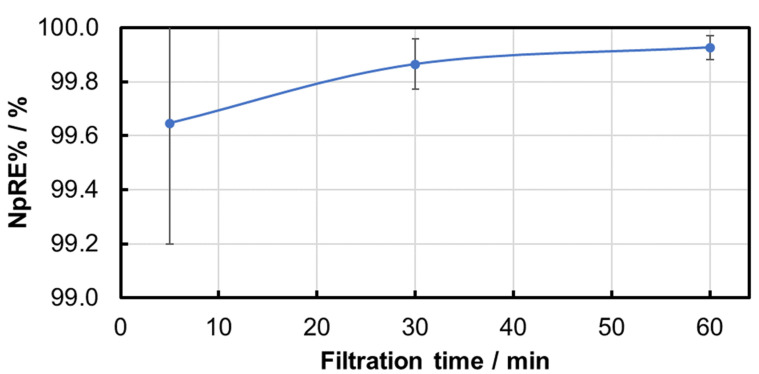
Time dependency of the average NpRE% and standard deviation during all PS MP filtration experiments.

In order to further examine the time dependency of the removal rate, an average value of the MRE% and NpRE% and the standard deviation were calculated for all filtration experiments at each of the three time points (Table 2, Figure 5 and Figure 6). A nearly complete removal of the particles was reached during these experiments. The very small variation in the results for all filtration experiments was confirmed by the very low standard deviation (Table 2).

**Figure 7:**
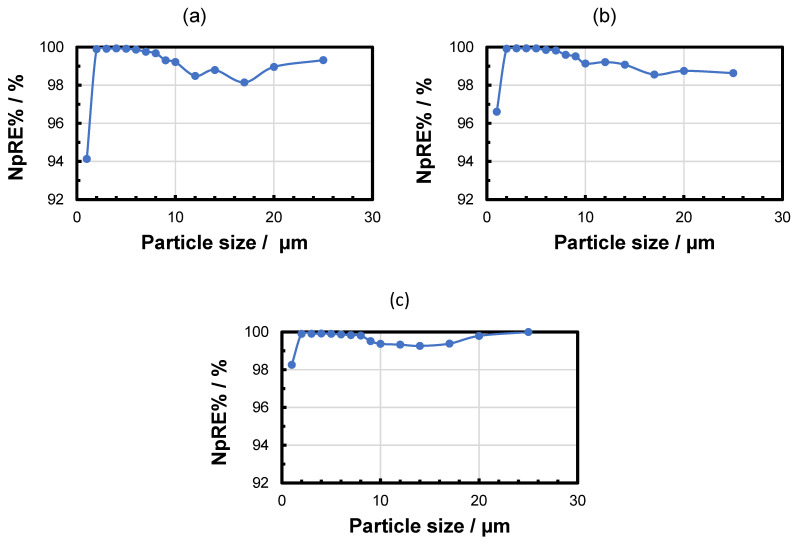
Time dependency of the NpRE% of different particle sizes during the PS MP filtration experiment at 0.2 bar after 5 min (**a**), 30 min (**b**), and 60 min (**c**).

In addition, the NpRE% is determined individually for each particle size class at each of the three measurement times in order to identify any difference in the separation of different particle sizes. The results confirmed that the removal rate for the small particles increased over time, which is characteristic of the buildup of a filter cake on the membrane. Small particles that were able to pass the nominal pore size at the beginning of the filtration were retained (by the filter cake) within the filtration duration. Larger particles were widely separated by the size effect. The number of larger particles (>10 µm) in the suspension was relatively low in comparison to the number of smaller particles in the suspension. Therefore, the passage of the membrane by a small number of larger particles could significantly influence the removal rate calculation. As an example, the particle size-dependent NpRE% for 0.2 bar pressure is shown in Figure 7.

**Figure 8:**
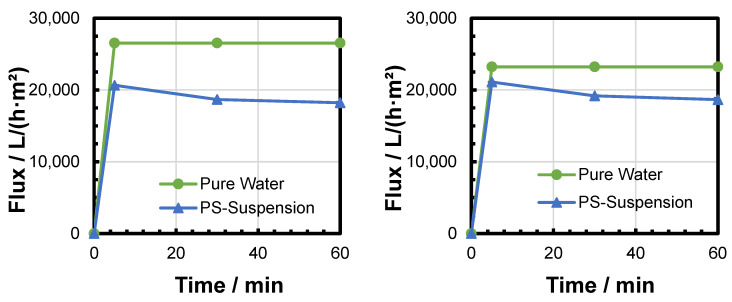
Evaluation of flux decline during two PS MP filtration experiments at 0.1 bar.

**Figure 9:**
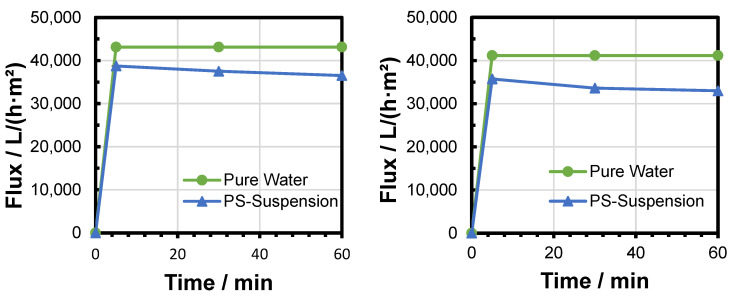
Evaluation of flux decline during two PS MP filtration experiments at 0.2 bar.

**Figure 10:**
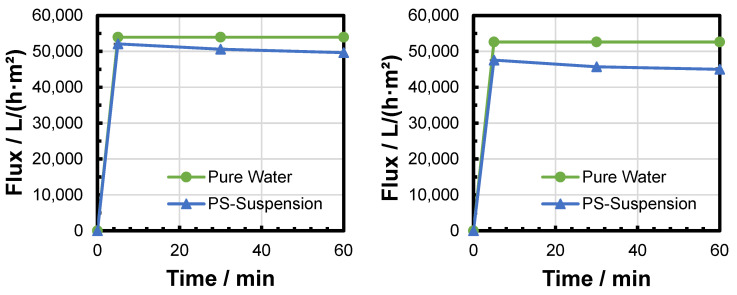
Evaluation of flux decline during two PS MP filtration experiments at 0.3 bar.

The flux was measured after different filtration durations and at different TMPs. The results of six filtration experiments were compared to the pure water flux of the respective fresh membrane in order to calculate the flux decline (Figure 8, Figure 9 and Figure 10). Increased TMP resulted in an increased flux. The flux decline over time during the filtration experiment was reduced after a short time (Table 3). Only a very small relative flux decline was observed between 30 and 60 min of filtration time. This observation could be attributed to a fast build-up of the filter cake (see Section 3.4. The difference in relative flux decline between 5 and 60 min decreased with increased pressure. This could be attributed to a faster deposition of particles within the first minutes of filtration due to the increased filtration pressure and elevated fluid velocity.

### 3.3. Backwashing Experiments

**Figure 11:**
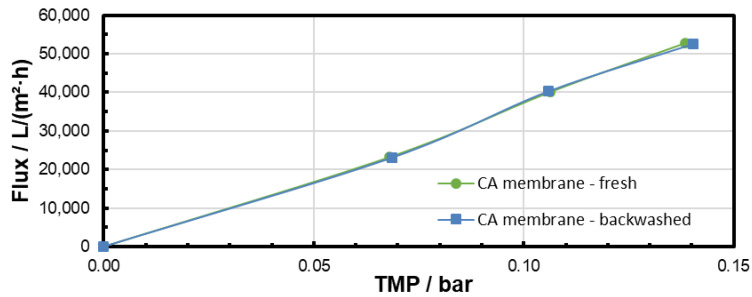
Pure water flux through the CA membrane before microfiltration and after 60 min microfiltration of the PS particle suspension at 0.1 bar and subsequent backwash.

**Figure 12:**
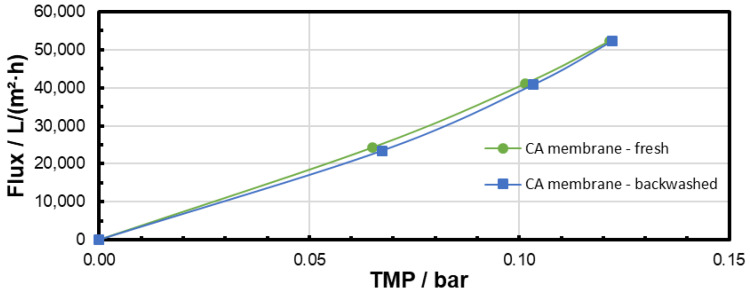
Pure water flux through the CA membrane before microfiltration and after 60 min microfiltration of the PS particle suspension at 0.2 bar and subsequent backwash.

**Figure 13:**
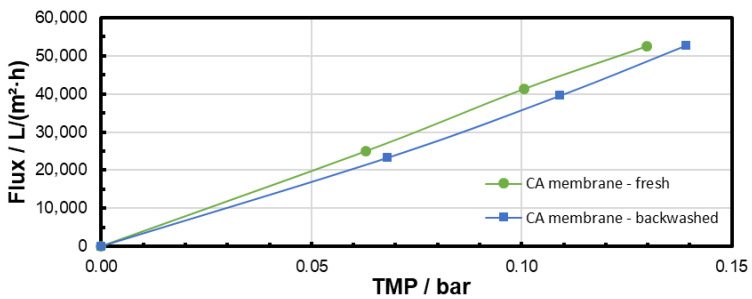
Pure water flux through the CA membrane before microfiltration and after 60 min microfiltration of the PS particle suspension at 0.3 bar and subsequent backwash.

The pure water flux was measured before microfiltration and after backwashing in order to observe the effectiveness of the backwash procedure. The results are presented in Figure 11, Figure 12 and Figure 13 and clearly confirm that particle fouling from microfiltration at low TMP could be removed by the backwashing procedure applied. The results also show that the flux was not completely recovered after microfiltration at 0.3 bar with the backwash procedure applied. This could be addressed to a difference in the particle-fouling mechanism dependent on the TMP (see Section 3.4).

### 3.4. Particle-Fouling Analysis

In order to further explain the particle-fouling mechanism, the used membranes were extensively analyzed using SEM, light microscopy, TGA, and water contact angle measurements.

### 3.5. Particle-Fouling Analysis by SEM and Light Microscopy

**Figure 14:**
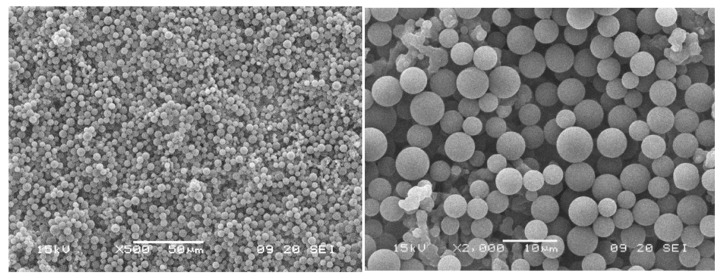

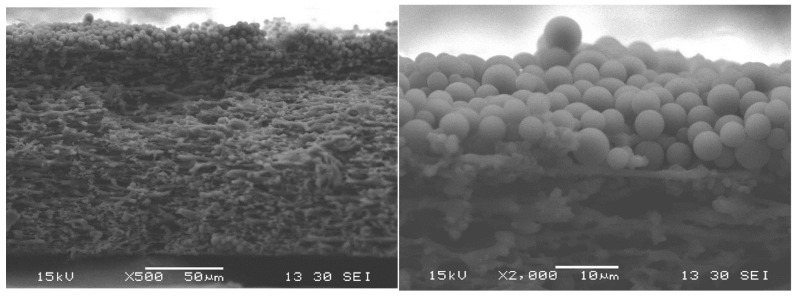
SEM image of the PS filter cake after 60 min filtration at 0.1 bar (top view and cross-section).

**Figure 15:**
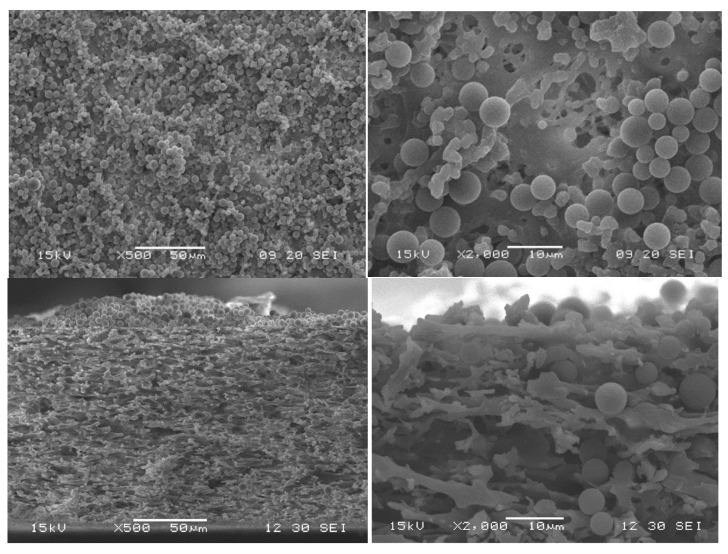
SEM image of the PS filter cake after 60 min filtration at 0.3 bar (top view and cross-section).

The build-up of a filter cake subsequent to the blockage (or partial closure) of pores is expected to describe the particle-fouling mechanism for many filtration processes [34,35,36]. Depending on the particle size and the membrane pore size, particle transport inside the pores can occur or not. This internal blockage of pores is not expected if the particles are larger than the pores. Our challenging microfiltration conditions (membrane pores and particle sizes on the same scale) enable the transport of particles inside larger pores. Therefore, the observation of the particle deposition in the pores, as well as the observation of the filter cake, gives indications of the fouling mechanics. PS microparticles can be assumed to be incompressible, and particle deformation can be excluded under the given conditions; however, particle movement within the filter cake, variation in the packaging, and changes in the cake height cannot be excluded and need to be identified for the microfiltration process [34,35,36]. SEM images of the membrane surface clearly show particle deposition during all TMPs (Figure 14 and Figure 15). The cross-sections are characterized by typical dead-end filter cakes. Increasing the TMP resulted in a decreased filter cake height. However, due to our analysis of the membrane surface, we could also identify more heterogeneous and irregular loading at increased TMP. This was observed, in particular, for the highest pressure applied (Figure 15). Increasing the TMP results in a decreased cake height, which is partly explained by filling voids with smaller particles at elevated TMP [35]. On the other hand, particles were also observed inside the membrane pores (Figure 15). Up to 99% of all particles smaller than the median pore size were already removed from the feed suspension after 5 min of filtration time. Due to our experiment design (particle and pore sizes) and in accordance with PMMA model filtration experiments by Dersoir et al. [37], we conclude that the smaller MP particles entered the pores and initiated an internal pore blockage, which led to a first flux decline. It is known that agglomerates were usually built up in the next blocking step [37]. Together with complete pore blockage by larger particles, this sequence initiated the build-up of the filter cake on the membrane surface. The number of particles inside the pores depends on the TMP. Elevated TMP resulted in an increased number of particles inside the pores. Agglomerates were only observed at the highest pressure tested (0.3 bar). However, the number of particles and agglomerates inside the pores was relatively low compared to the number of particles on the membrane surface, which led to the assumption that most of the small particles and agglomerates were already flushed out of the pores by water after a short time. It is very probable that this small amount of small particles was deposited on the filter cake within the duration of the experiment.

**Figure 16:**
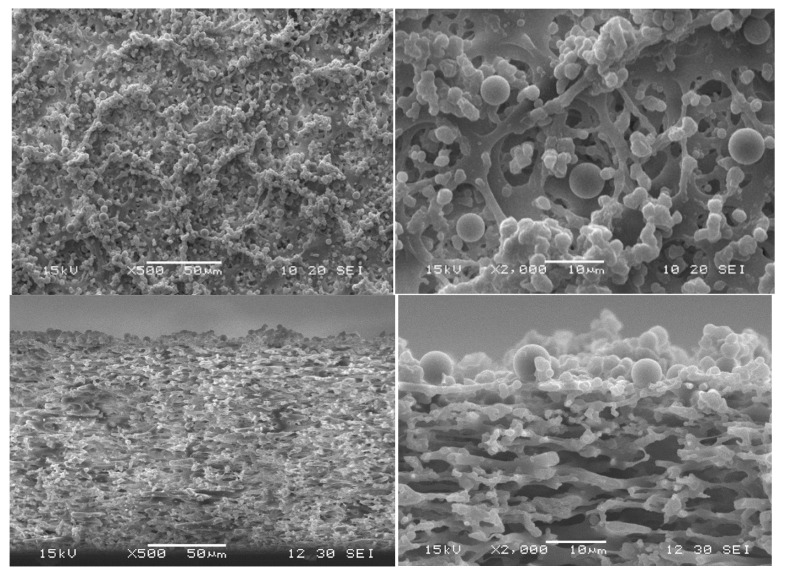
SEM image of the PS filter cake after 60 min filtration at 0.1 bar and subsequent backwashing (top view and cross-section).

**Figure 17:**
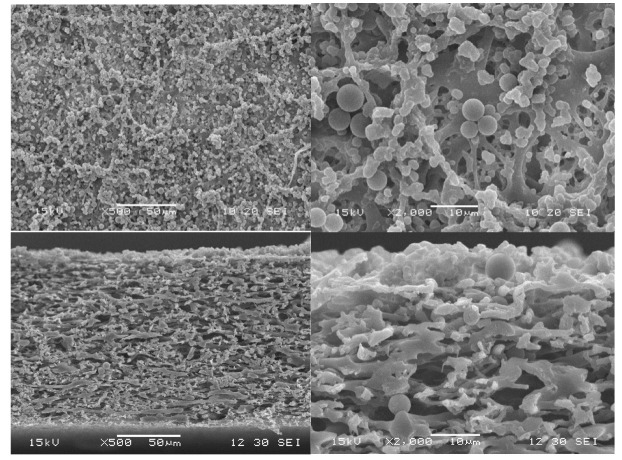
SEM image of the PS filter cake after 60 min filtration at 0.3 bar and subsequent backwashing (top view and cross-section).

Almost all of the filter cake was removed by backwashing (Figure 16 and Figure 17). Due to the low forces between MP particles and the membrane, the cleaning by backwash is very efficient. Only isolated particles remained inside the membrane, which also confirmed the low interparticle forces within the agglomerates inside the pores.

The pore-blocking procedure, cake morphology (structure), and cake size (height) are decisive factors for the efficiency of the backwashing procedure. The experiments of backwashing after filtration of identical MP suspensions at different TMPs show that more heterogeneous cake morphologies and a higher number of blocked pores after filtration impede the backwashing. Especially for the highest TMP, flux recovery was not completely possible after the backwash (see Figure 13 and Figure 17). However, for all TMPs, the flux recovery clearly confirmed that backwashing is an efficient MP particle-fouling cleaning technology at our experimental conditions.

### 3.6. Particle-Fouling Analysis by TGA

**Figure 18:**
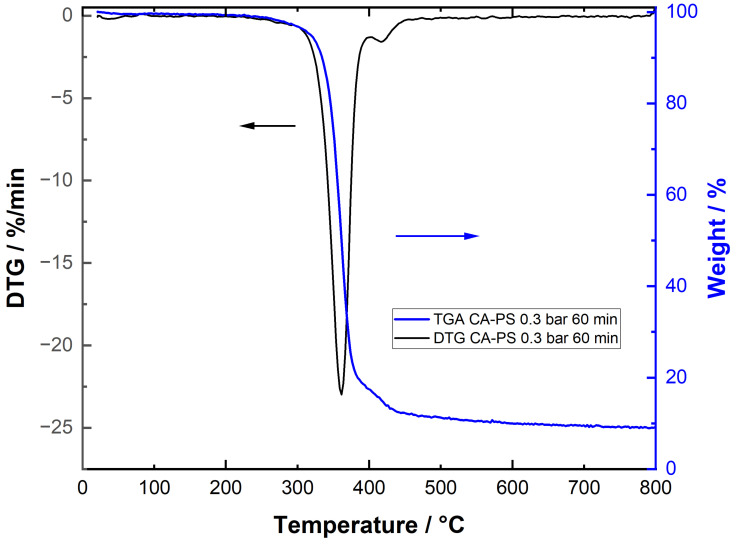
TGA and DTG signals of the CA membrane after 60 min microfiltration of the PS suspension at 0.3 bar.

The TG analysis of a CA membrane after a 60 min PS microfiltration using 0.3 bar filtration pressure is shown in Figure 18. As expected, the CA membrane decomposed in the temperature range 280–380 °C (first weight loss step). Afterwards, a second decomposition step was clearly observed, which is characteristic of the decomposition of the deposited PS between ~390 and 430 °C. The DTG graphs confirm the existence of two decomposition steps, which can be attributed to membrane (CA) and particle foulant/filter cake (PS).

**Figure 19:**
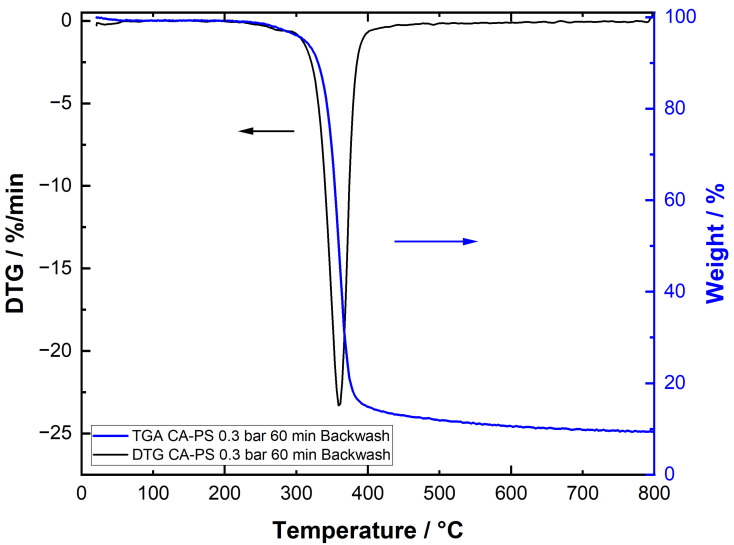
TGA and DTG signal of the CA membrane after 60 min microfiltration of PS suspension at 0.3 bar and subsequent backwashing procedure.

For comparison, a TG analysis was also performed for a membrane after a 60 min PS filtration at 0.3 bar and subsequent backwashing. No PS signal was monitored during this measurement. Only one peak (CA) was observed during the DTG, which also indicates that most of the PS was removed during the backwashing (Figure 19).

Due to inhomogeneous particle deposition and complex sample preparation, it is hard to provide a statement on whether TGA is suitable for the analysis of MP particle fouling on CA membranes. Our results show that TGA is suitable for the detection of MP on CA membranes. We also came to the conclusion that accurate sample preparation and detailed knowledge about the behavior of the included materials at process conditions are decisive for the usage of TGA for fouling analysis. Partly overlapping residual decomposition of small amounts cannot be excluded (see DTG analysis of pure materials in Supplementary Information). In comparison to our clean lab experiments, combinations of different MPs will be filtered in the field. This will increase the number of degrees of freedom and will complicate the TG analysis. On the other hand, the different decomposition temperatures of CA and most MP foulants could be a key for MP detection and identification on the membrane. Other analytical methods may hold advantages for extensive characterization.

### 3.7. Particle-Fouling Analysis by Water Contact Angle Measurements

**Figure 20:**
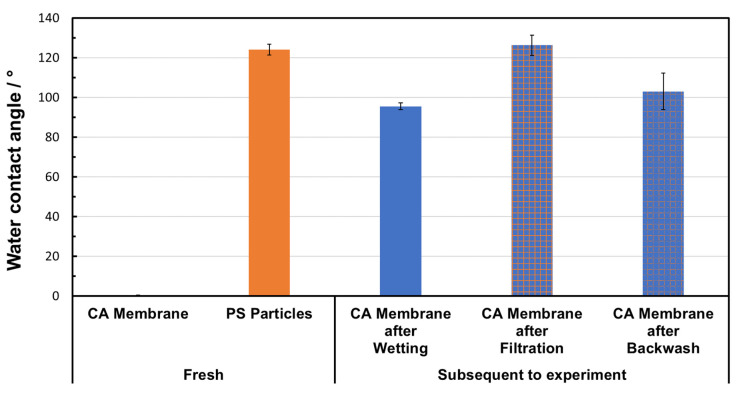
Results of water contact angle measurements for fresh materials and the used CA membrane.

The results of the water contact angle measurement are presented in Figure 20. The fresh CA membrane was characterized by a water contact angle of 0°, indicating a hydrophilic surface, which is consistent with values reported in the literature [20,38]. After wetting and subsequent drying, the contact angle increased to 96 ± 2°, which indicates that the original hydrophilicity was reduced by this treatment. A significantly higher contact angle of 124 ± 3° was measured for the PS powder, which is clearly attributed to its hydrophobic properties. After filtration, the membranes were characterized by a contact angle of 126 ± 5°, which is almost identical to that of the PS powder. It can be concluded that the top layer of PS particles completely covered the membrane and that its hydrophobic properties dominated on the surface. After backwashing, the contact angle dropped to 103 ± 9°, which is between the values of the membranes after wetting and after filtration. This indicates that most of the PS layer is removed by backwashing, but the membrane does not completely return to its original state. The high standard deviation also shows that the cleaning was carried out rather unevenly, so that inhomogeneous residues remain on the membrane. Overall, the measured values show that the filtration resulted in a hydrophobic PS top layer, which was only partially removed by backwashing. The membrane was not completely regenerated at the selected backwashing conditions.

PS particles and fresh, water-treated, and fouled membranes were also analyzed using ATR-FTIR. However, due to the overlapping of the characteristic peaks, no clear detection of PS fouling on CA was possible with ATR-FTIR. The results are provided in the Supplementary Information.

## 4. Discussion and Conclusions

The focus of this study was to experimentally investigate the microfiltration characteristics of a commercial CA microfiltration membrane for the removal of PS microplastic particles from water during dead-end operation with a special view on particle size aspects. A comparable membrane pore size/PS particle size combination was selected in order to challenge the microfiltration. The particle fouling mechanism was investigated by different methods. Backwashing efficiency experiments round off the investigations.

Within our experiments, most of the microplastic particles were already removed from the feed suspension after a short microfiltration duration. The removal efficiency was particle size-dependent, and especially small particles were further reduced during the ongoing microfiltration, while the larger particles were already removed within the first minutes of microfiltration. This observation was attributed to the pore blocking (internal and/or complete) and the build-up of the filter cake. Increased filtration pressure resulted in decreased flux decline, which was attributed to more heterogeneous particle layer formation at elevated pressure. On the other hand, a thinner filter cake height and an increased number of particles within the membrane pores were detected at elevated pressure. This difference in particle fouling structure at low and elevated filtration pressure significantly influences the backwashing efficiency. The particle fouling resulting from low-pressure filtration was almost completely removed due to the backwashing procedure applied, while an increased filtration pressure resulted in a different particle fouling structure, which negatively influenced the backwashing efficiency at given conditions.

Microfiltration could be a decisive technology for the removal of MP from, e.g., industrial process wastewater or within the purification of drinking water. The results of our lab-scale investigations show that material characteristics, particle size and pore size, and process conditions influence the MP removal and backwashing efficiency. Knowledge on MP–membrane interactions and resulting particle-fouling mechanisms is a decisive key for effective MP removal and is a prerequisite for further process development. For example, a subsequent treatment of the concentrated MP backwash solution could be sedimentation, precipitation, additional filtration, concentrating, or further conditioning steps.

The spherical microplastic particles are well suited for our investigation. However, microplastics also appear in different morphologies, e.g., fibers or films. Therefore, we will also take into account the particle morphology within our future investigations. A lab-scale dead-end filtration mode was addressed during the investigations presented in this paper. For many water purification applications, cross-flow filtration is very common on an industrial scale. Therefore, future examinations will also focus on cross-flow mode in order to compare the microfiltration and particle-fouling characteristics, which is a prerequisite for process development.

## Figures and Tables

**Table 1:** Characteristics of the PS microplastic water feed suspensions.

Suspension ID	Average Particle Size/µm	Standard Deviation of Particle Size Distribution/µm	Turbidity/NTU
1	3.20	1.60	2.74 ± 0.04
2	3.02	1.41	2.52 ± 0.04
3	3.20	1.71	2.92 ± 0.03
4	3.11	1.60	2.83 ± 0.04
5	3.12	1.58	3.09 ± 0.09
6	3.00	1.36	2.71 ± 0.02

**Table 2:** Median particle size of PS microplastic in suspension and removal efficiency with standard deviation during all MP filtration experiments.

Sample	Median Particle Size/µm	MRE%	NpRE%
Feed suspension	3.00	/	/
Filtrate after 5 min	2.00	99.56 ± 0.27	99.65 ± 0.45
Filtrate after 30 min	2.00	99.76 ± 0.14	99.87 ± 0.09
Filtrate after 60 min	2.33	99.84 ± 0.11	99.93 ± 0.04

**Table 3:** Relative flux decline for PS suspension microfiltration experiments at different filtration pressures.

Filtration Experiment	Pressure/bar	Flux Decline After 5 min/%	Flux Decline After 30 min/%	Flux Decline After 60 min/%
Filtration 1	0.1	−22.2	−29.7	−31.4
Filtration 2	0.1	−9.1	−17.4	−19.7
Filtration 3	0.2	−10.2	−13.1	−15.4
Filtration 4	0.2	−13.3	−18.4	−19.9
Filtration 5	0.3	−3.4	−6.2	−8.0
Filtration 6	0.3	−9.6	−13.2	−14.5

## Data Availability

Data are contained within the article or within the Supplementary Material. Further inquiries can be directed to the corresponding author.

## Supplementary Materials

The following supporting information can be downloaded at https://www.mdpi.com/article/10.3390/membranes15090272/s1, Figure S1. SEM images of PS particles; Figure S2. Light microscopy (left) and SEM images (middle, right) of fresh CA membrane; Figure S3. Light microscopy: PS filter cake after 60 min filtration at 0.1 bar (left), membrane surface after subsequent backwashing (right); Figure S4. Light microscopy: PS filter cake after 60 min filtration at 0.2 bar (left), membrane surface after subsequent backwashing (right); Figure S5. Light microscopy: PS filter cake after 60 min filtration at 0.3 bar (left), membrane surface after subsequent backwashing (right); Figure S6. SEM image of the PS filter cake after 60 min filtration at 0.2 bar (top view); Figure S7. SEM image of the PS filter cake after 60 min filtration at 0.2 bar and subsequent backwashing (top view); Figure S8. SEM image of the PS filter cake after 60 min filtration at 0.2 bar (cross section); Figure S9. SEM image of the PS filter cake after 60 min filtration at 0.2 bar and subsequent backwashing (cross section); Figure S10. FTIR spectra of CA membrane, CA membrane after PS filtration, CA membrane after PS filtration and subsequent backwashing and FTIR spectrum of PS particles; Figure S11. TGA and DTG signals of the CA membrane after 60 min flushing clean H2O at 0.3 bar; Figure S12. TGA and DTG signals of the PS particles after treatment in H2O.

## References

[1] Tajwara M., Gazi M.J., Sahaa S.K. (2021). Characterization and Spatial Abundance of Microplastics in the Coastal Regions of Cox’s Bazar, Bangladesh: An Integration of Field, Laboratory, and GIS Techniques. Soil Sediment Contam. Int. J..

[2] Park H., Park B. (2021). Review of Microplastic Distribution, Toxicity, Analysis Methods, and Removal Technologies. Water.

[3] Xu Y., Ou Q., Wang X., van der Hoek J.P., Liu G. (2024). Mass Concentration and Removal Characteristics of Microplastics and Nanoplastics in a Drinking Water Treatment Plant. ACS EST Water.

[4] Ma B., Xue W., Ding Y., Hu C., Liu H., Qu J. (2019). Removal characteristics of microplastics by Fe-based coagulants during drinking water treatment. J. Environ. Sci..

[5] Acarer S. (2023). A review of microplastic removal from water and wastewater by membrane technologies. Water Sci. Technol..

[6] Di Bella G., Corsino S.F., De Marines F., Lopresti F., La Carrubba V., Torregrossa M., Viviani G. (2022). Occurrence of Microplastics in Waste Sludge of Wastewater Treatment Plants: Comparison between Membrane Bioreactor (MBR) and Conventional Activated Sludge (CAS) Technologies. Membranes.

[7] Egea-Corbacho A., Martín-García A.P., Franco A.A., Quiroga J.M., Andreasen R.R., Jørgensen M.K., Christensen M.L. (2023). Occurrence, identification and removal of microplastics in a wastewater treatment plant compared to an advanced MBR technology: Full-scale pilot plant. J. Environ. Chem. Eng..

[8] Rius-Ayra O., Biserova-Tahchieva A., LLorca-Isern N. (2021). Surface-functionalised materials for microplastic removal. Mar. Pollut. Bull..

[9] Murray A., Örmeci B. (2020). Removal Effectiveness of Nanoplastics (<400 nm) with Separation Processes Used for Water and Wastewater Treatment. Water.

[10] Akrsu C., Kumbur H., Kideys A.E. (2021). Removal of microplastics from wastewater through electrocoagulation-electroflotation and membrane filtration processes. Water Sci. Technol..

[11] Ali I., Tan X., Mustafa G., Gao J., Peng C., Naz I., Duan Z., Zhu R., Ruan Y. (2024). Removal of micro- and nanoplastics by filtration technology: Performance and obstructions to market penetrations. J. Clean. Prod..

[12] Kundu A., Shetti N.P., Basu S., Reddy R.R., Nadagouda M.N., Aminabhavi T.M. (2021). Identification and removal of micro- and nano-plastics: Efficient and cost-effective methods. Chem. Eng. J..

[13] Li J., Wang B., Chen Z., Ma B., Chen J.B. (2021). Ultrafiltration membrane fouling by microplastics with raw water: Behaviors and alleviation methods. Chem. Eng. J..

[14] Golgoli M., Khiadani M., Shafieian A., Sen T.K., Hartanto Y., Johns M.L., Zargar M. (2021). Microplastics fouling and interaction with polymeric membranes: A review. Chemosphere.

[15] Shen M., Zhao Y., Liu S., Hu T., Zheng K., Wang Y., Lian J., Meng G. (2023). Recent advances on micro/nanoplastic pollution and membrane fouling during water treatment: A review. Sci. Total Environ..

[16] Ghasemi S., Yan B., Zargar M., Ling N.N.A., Fridjonsson E.O., Johns M.L. (2023). Impact of microplastics on organic fouling of hollow fiber membranes. Chem. Eng. J..

[17] Enfrin M., Wang J., Merenda A., Dumèe L.F., Lee J. (2021). Mitigation of membrane fouling by nano/microplastics via surface chemistry control. J. Membr. Sci..

[18] Enfrin M., Lee J., Fane A.G., Dumée L.F. (2021). Mitigation of membrane particulate fouling by nano/microplastics via physical cleaning strategies. Sci. Total Environ..

[19] Pizzichetti A.R.P., Pablos C., Alvarez-Fernandez C., Reynolds K., Stanley S., Marugan J. (2021). Evaluation of membranes performance for microplastic removal in a simple and low-cost filtration system. J. Environ. Chem. Eng..

[20] Pizzichetti A.R.P., Pablos C., Alvarez-Fernandez C., Reynolds K., Stanley S., Marugan J. (2023). Kinetic and mechanistic analysis of membrane fouling in microplastics removal from water by dead-end microfiltration. J. Environ. Chem. Eng..

[21] Slejko E.A., Tuan A., Scuor N. (2024). From waste to value: Characterization of recycled cellulose acetate for sustainable waste management. Waste Manag. Bull..

[22] Islam M.D., Uddin F.J., Rashid T.U., Shahruzzaman M. (2023). Cellulose acetate-based membrane for wastewater treatment—A state-of-the-art review. Mater. Adv..

[23] Peonemann K.-V., Nunes S.P. (2010). Membranes for Water Treatment.

[24] Zhao X., Zhanga R., Liua Y., Hea M., Sua Y., Gaob C., Jianga Z. (2018). Antifouling membrane surface construction: Chemistry plays a critical role. J. Membr. Sci..

[25] Tay J.-H., Liu J., Sun D.D. (2003). Quantification of membrane fouling using thermogravimetric method. J. Membr. Sci..

[26] Kamarudin D., Hashim N.A., Ong B.H., Faried M., Suga K., Umakoshi H., Mahari W.A.W. (2022). Alternative fouling analysis of PVDF UF membrane for surface water treatment: The credibility of silver nanoparticles. J. Membr. Sci..

[27] da Conceicao M., Lucena C., de Alencar A.E.V., Mazzeto S.E., Soares S.A. (2003). The effect of additives on the thermal degradation of cellulose acetate. Polym. Degrad. Stab..

[28] Shaikh H.M., Anis A., Poulose A.M., Al-Zahrani S.M., Madhar N.A., Alhamidi A., Aldeligan S.H., Alsubaie F.S. (2022). Synthesis and Characterization of Cellulose Triacetate Obtained from Date Palm (*Phoenix dactylifera* L.) Trunk Mesh-Derived Cellulose. Molecules.

[29] Marcilla A., Beltrán M. (1995). Kinetic Study of the thermal decomposition of polystyrene and polyethylene-vinyl acetate graft copolymers by thermogravimetric analysis. Polym. Degrad. Stab..

[30] Faravelli T., Pinciroli M., Pisano F., Bozzano G., Dente M., Ranzi E. (2001). Thermal degradation of polystyrene. J. Anal. Appl. Pyrolysis.

[31] Jang B.N., Wilkie C.A. (2005). The thermal degradation of polystyrene nanocomposite. Polymer.

[32] Corami F., Rosso B., Bravo B., Gambaro A., Barbante C. (2020). A novel method for purification quantitative analysis and characterization of microplastic fibers using Micro-FTIR. Chemosphere.

[33] Yadav N., Hakkarainen M. (2022). Degradation of Cellulose Acetate in Simulated Aqueous Environments: One-Year Study. Macromol. Mater. Eng..

[34] Mackley M.R., Sherman N.E. (1992). Cross-Flow Cake Filtration Mechanism and Kinetics. Chem. Eng. Sci..

[35] Vyasa H.K., Mawsona A.J., Bennett R.J., Marshall A.D. (2000). A new method for estimating cake height and porosity during crossflow filtration of particulate suspensions. J. Membr. Sci..

[36] Chellam S., Xu W. (2006). Blocking laws analysis of dead-end constant flux microfiltration of compressible cakes. J. Colloid Interface Sci..

[37] Dersoir B., Schofield A.B., Robert de Saint Vincent M., Tabuteau H. (2019). Dynamics of pore fouling by colloidal particles at the particle level. J. Membr. Sci..

[38] Ahmad A.L., Mat Yasin N.H., Derek C.J.C., Lim J.K. (2013). Microfiltration of Chlorella sp.: Influence of material and membrane pore size. Membr. Water Treat..

