# Supplementary material for: Investigations on the Particle Fouling and Backwash Efficiency During Microplastic Microfiltration–Particle Size Aspects
Source: Membranes (Basel). 2025 Sep 9;15(9):272. doi: 10.3390/membranes15090272 (PMC12471478; doi:10.3390/membranes15090272)
Supplement: Supplementary file 1 [file membranes-15-00272-s001.zip › membranes-3753956-supplementary.pdf]

Supplementary Material

Investigations on the Particle Fouling and Backwash Efficiency During Microplastic Microfiltration–Particle Size Aspects

Saeedeh Saremi [1,2], Leonie Marie Scheer [1], Gerhard Braun [1], Marcus Koch [1], Markus Gallei [2,3] and Matthias Faust [1,*]

[1] Institute for Physical Process Technology, Saarland University of Applied Sciences, 66117 Saarbrücken, Germany; saeedeh.saremi@htwsaar.de (S.S.); gerhard.braun@htwsaar.de (G.B.); marcus.koch@htwsaar.de (M.K.)
[2] Polymer Chemistry, Saarland University, 66123 Saarbrücken, Germany; markus.gallei@uni-saarland.de
[3] Saarene, Saarland Center for Energy Materials and Sustainability, Saarland University, 66123 Saarbrücken, Germany
[*] Correspondence: matthias.faust@htwsaar.de

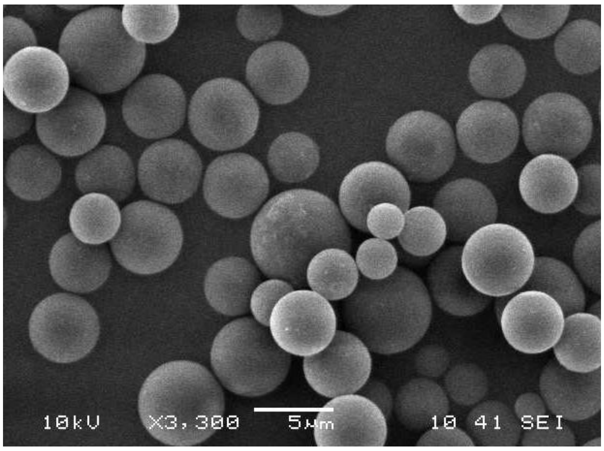

Figure S1. SEM images of PS particles.

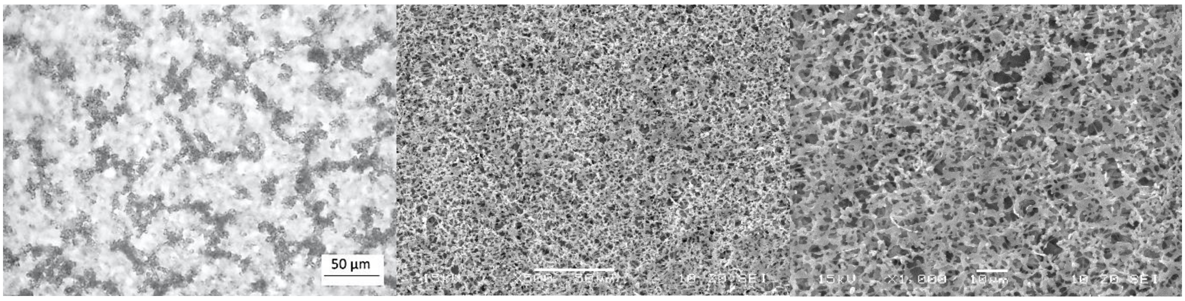

Figure S2. Light microscopy (left) and SEM images (middle, right) of fresh CA membrane.

Calculation of the removal efficiency:

The number particle removal efficiency NpRE% was calculated from the particle size measurements. Therefore, the total number of particles in the feed suspension in the beginning

of an experiment $n_{Total,Start}$ and the number of particles in the filtrate after defined filtration time $n_{Total,Fil,t}$ were used in the following equation:

$$NpRE\% = \left(1 - \frac{n_{Total,Fil,t}}{n_{Total,Start}}\right) \cdot 100\ \%$$

The NpRE% was also calculated for specific particle sizes with the number of particles of a specific particle size in the feed suspension in the beginning of an experiment and with the number of particles with the same specific particle size in the filtrate after defined filtration time.

The mass removal efficiency MRE% was calculated from the particle mass in the feed suspension in the beginning of an experiment $m_{MP,Start}$ and the particle mass in the filtrate after defined filtration time $m_{MP,Fil,t}$ for spherical particles.

$$MRE\% = \left(1 - \frac{m_{MP,Fil,t}}{m_{MP,Start}}\right) \cdot 100\ \%$$

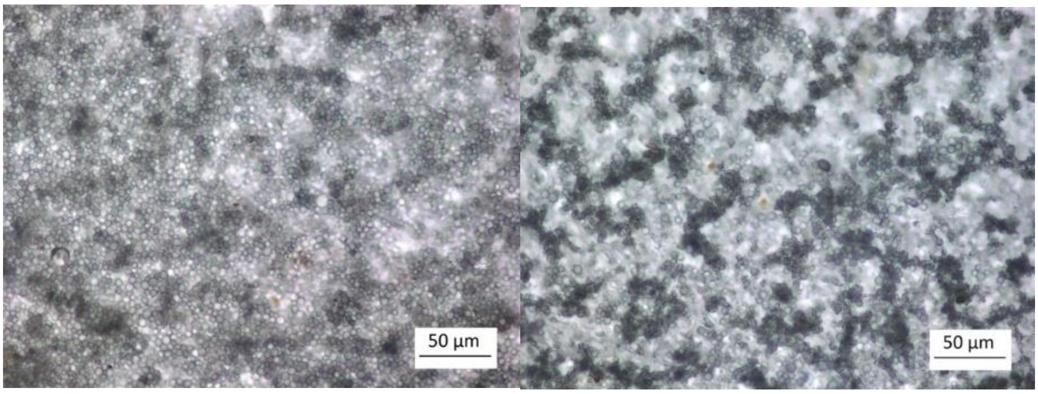

Figure S3. Light microscopy: PS filter cake after 60 min filtration at 0.1 bar (left), membrane surface after subsequent backwashing (right).

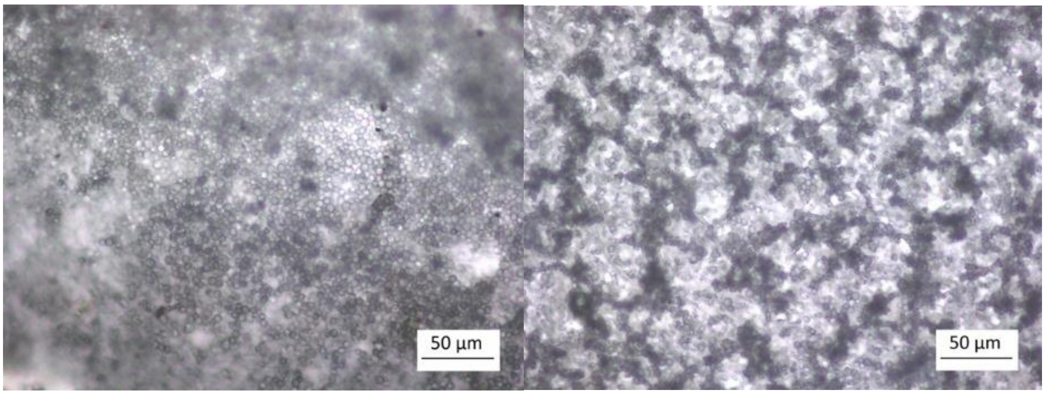

Figure S4. Light microscopy: PS filter cake after 60 min filtration at 0.2 bar (left), membrane surface after subsequent backwashing (right).

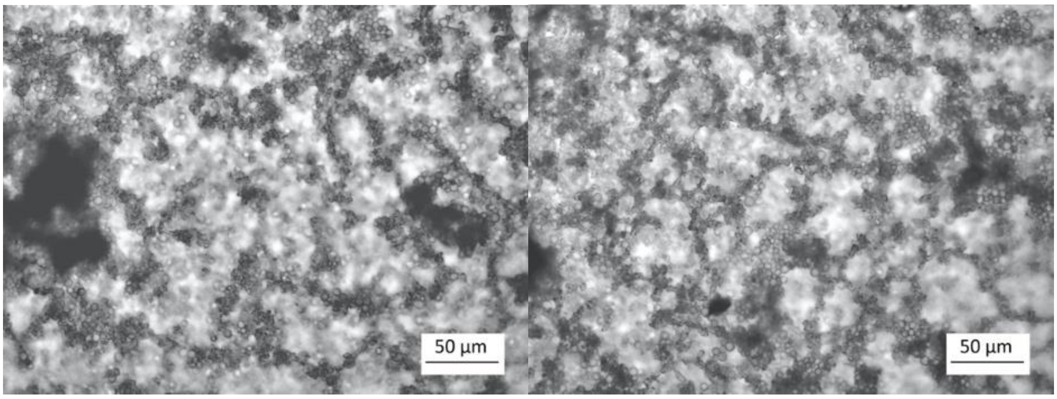

Figure S5. Light microscopy: PS filter cake after 60 min filtration at 0.3 bar (left), membrane surface after subsequent backwashing (right).

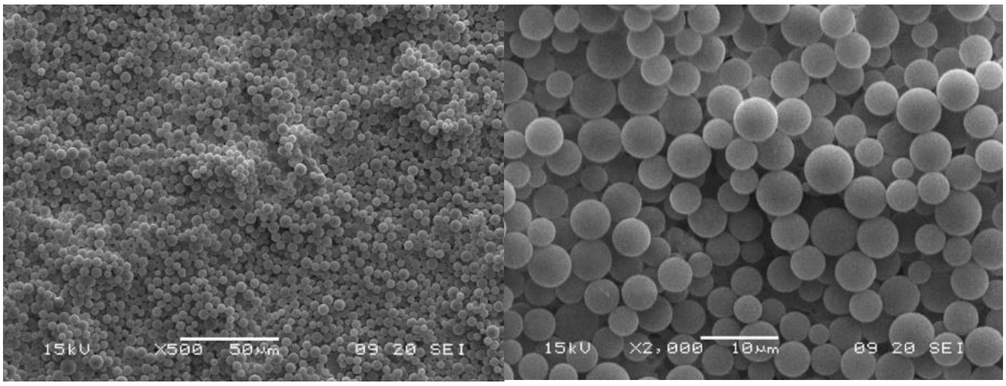

Figure S6. SEM image of the PS filter cake after 60 min filtration at 0.2 bar (top view).

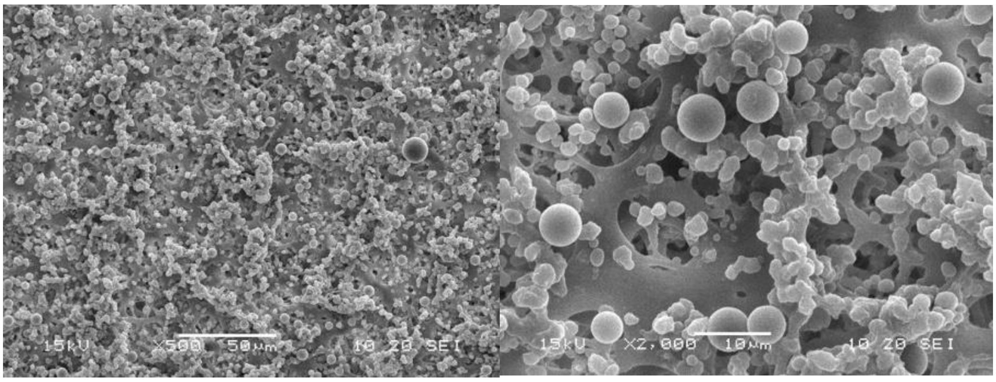

Figure S7. SEM image of the PS filter cake after 60 min filtration at 0.2 bar and subsequent backwashing (top view).

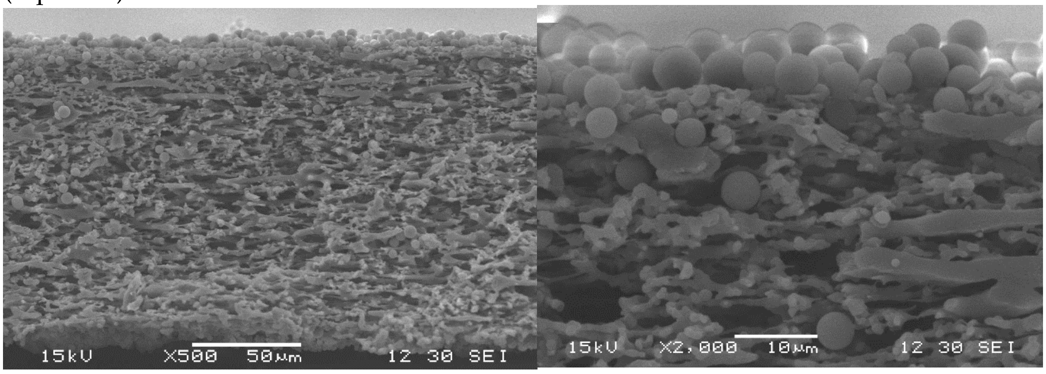

Figure S8. SEM image of the PS filter cake after 60 min filtration at 0.2 bar (cross section).

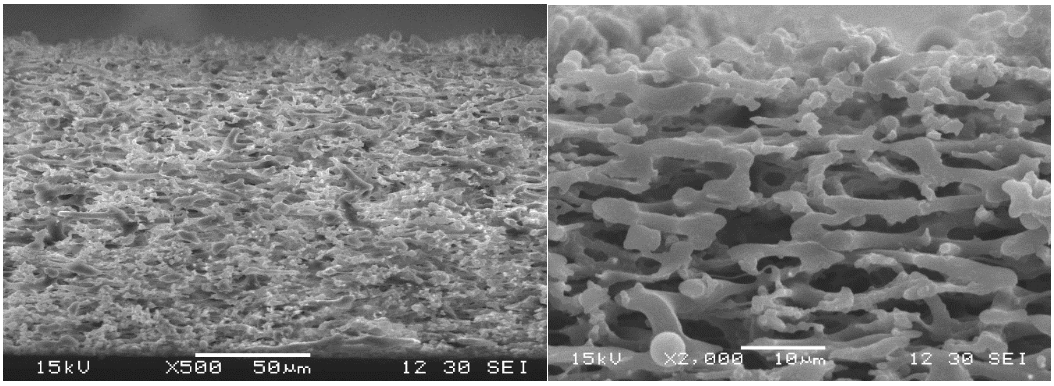

Figure S9. SEM image of the PS filter cake after 60 min filtration at 0.2 bar and subsequent backwashing (cross section).

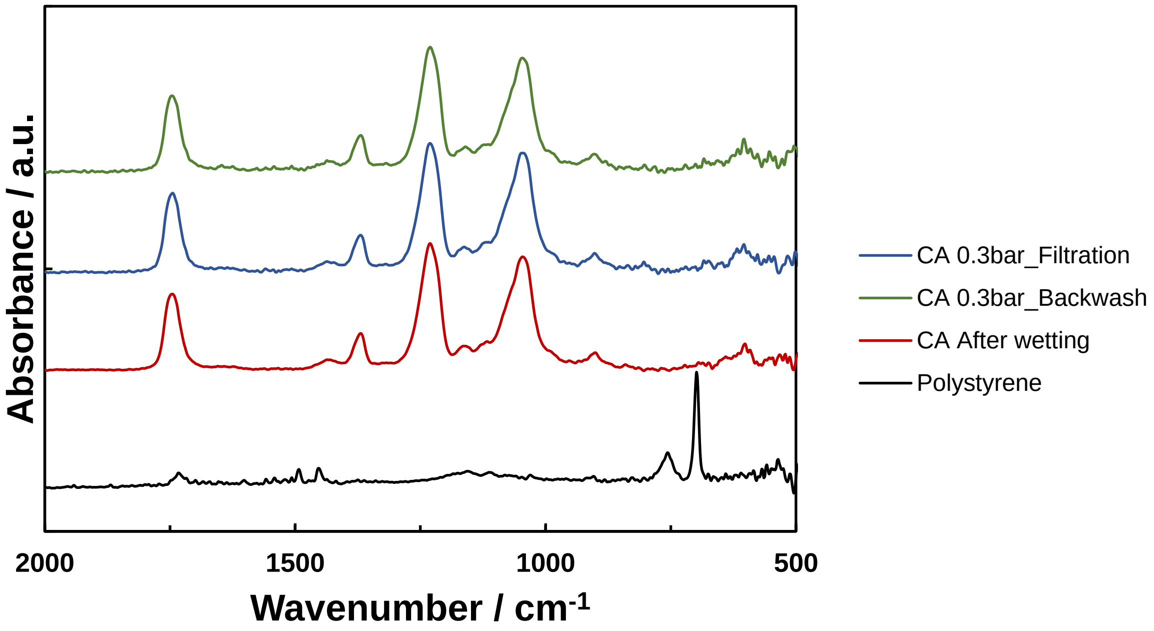

Figure S10. FTIR spectra of CA membrane, CA membrane after PS filtration, CA membrane after PS filtration and subsequent backwashing and FTIR spectrum of PS particles.

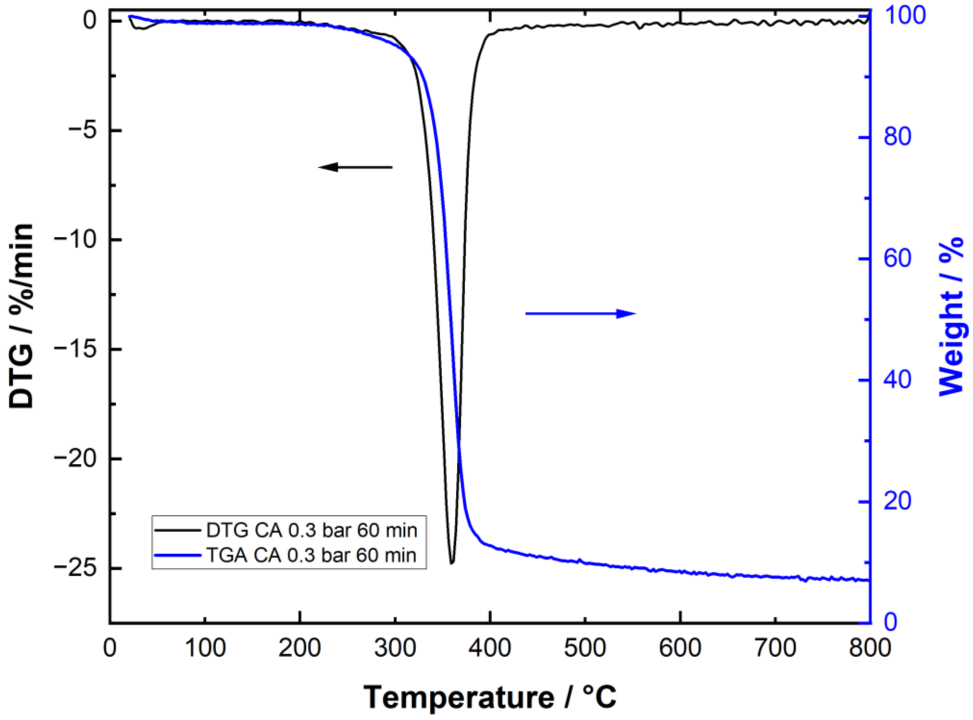

Figure S11. TGA and DTG signals of the CA membrane after 60 min flushing clean H_2O at 0.3 bar.

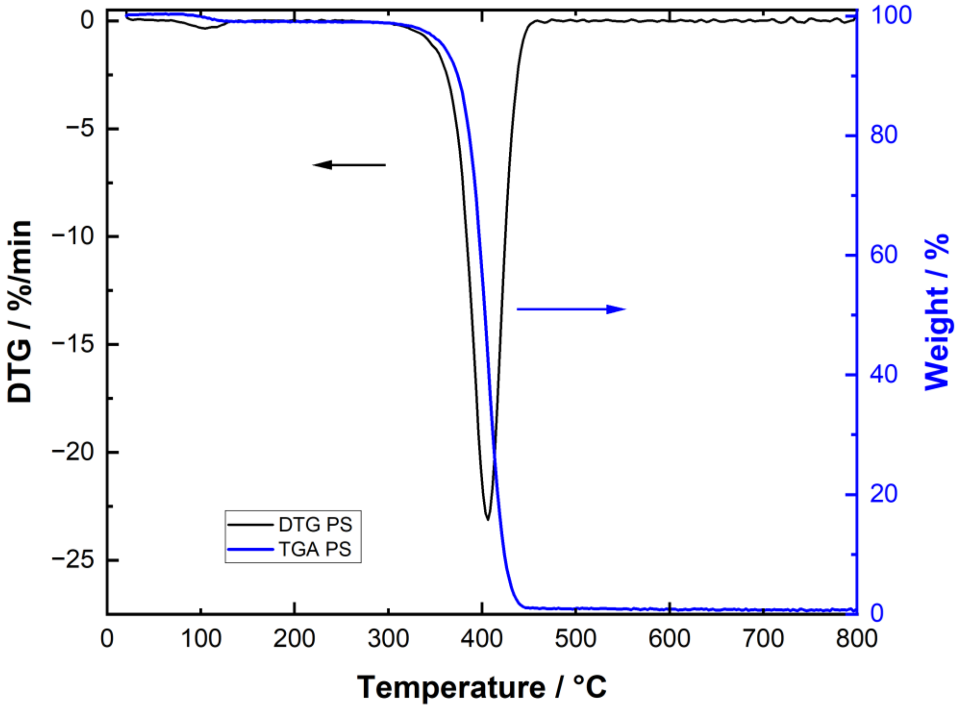

Figure S12. TGA and DTG signals of the PS particles after treatment in H_2O.
